# Complete Atrioventricular Block Caused by Retrograde Transaortic Approach

**DOI:** 10.3390/jcdd9090293

**Published:** 2022-09-03

**Authors:** Songwen Chen, Xiaofeng Lu, Qitong Zhang, Yong Wei, Genqing Zhou, Shaowen Liu

**Affiliations:** Department of Cardiology, Shanghai General Hospital, Shanghai Jiao Tong University School of Medicine, Shanghai 200080, China

**Keywords:** atrioventricular block, catheter ablation, retrograde transaortic approach, premature ventricular complex

## Abstract

A 61-year-old female was referred for catheter ablation of symptomatic and frequent premature ventricular complexes presented with right bundle branch block and a prominent inferior frontal plane QRS axis. A retrograde transaortic approach was routinely performed. A sustained complete atrioventricular block was repeatedly encountered while the ablation catheter was attempting to cross the aortic valve with different curves and manipulations. The procedure was abandoned. The mechanical atrioventricular block could only have been caused by the retrograde transaortic approach. We should be cautious when performing a retrograde transaortic catheter manipulation in some patients.

## 1. Introduction

Radiofrequency ablation has been demonstrated to be a low-risk and effective treatment for eliminating premature ventricular complexes (PVC), even those originated from the proximal left anterior fascicle [1,2]. Due to the anatomic characteristics, the ablation of those arrhythmias may potentially increase the risk of injuring the special conduction system, such as atrioventricular (AV) block, left bundle branch block, etc. The retrograde transaortic approach is a widely used access route for the mapping and ablation of ventricular arrhythmias arising from the left ventricular endocardium, including PVC originated from the left anterior fascicle [1,2,3]. The retrograde transaortic approach was safe and useful in our previous clinical practice [1,2]. It has been reported that vascular complications were more frequent with the retrograde transaortic approach [3,4]. Moreover, it has been reported that some complications were associated with the retrograde transaortic approach, including iatrogenic aortic dissection, coronary damage, valve leaflets damage and so on [3]. However, a retrograde transaortic approach associated with a complete atrioventricular block has rarely been reported. Here, we present a case of sustained complete atrioventricular block caused by only a retrograde transaortic approach but not by catheter ablation. 

## 2. Case Report

**Figure 1:**
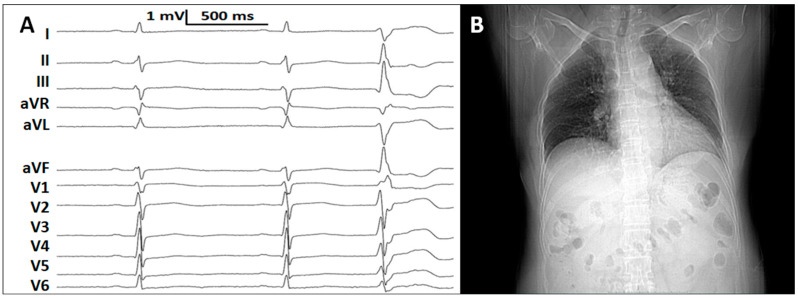
The ECG and chest radiograph before procedure. (**A**) A 12-lead ECG showing the normal sinus rhythm (with normal PR interval of 168 ms) and premature ventricular complexes (PVC). The QRS duration of the sinus rhythm and PVC was 97 ms and 124 ms, respectively. (**B**) Chest radiograph indicating a horizontal heart with a cardiothoracic ratio of 0.58.

A 61-year-old female (body height 1.70 m, body weight 72 kg) was referred for radiofrequency catheter ablation of PVC. She had a history of symptomatic frequent PVC, which had been refractory to bisoprolol, verapamil, and mexiletine, for 2 years. Her PVC count was 13,038 beats (with a burden of 13.0%) recorded by a 24 h Holter. The QRS duration of the PVC was 124 ms, longer than that of the sinus rhythm (97 ms, Figure 1A). The QRS morphology of the PVC showed a right bundle branch block and a prominent inferior frontal plane QRS axis. No preexisting conduction defect was detected by ECG and Holter. She also had a history of hypertension well-controlled by amlodipine, and a history of surgery for bilateral breast cancer and pituitary adenoma. Chest radiograph indicated a horizontal heart with a cardiothoracic ratio of 0.58 (Figure 1B). Echocardiography showed normal cardiac diameters and function. No evidence of structural heart disease was revealed by routine examinations. 

**Figure 2:**
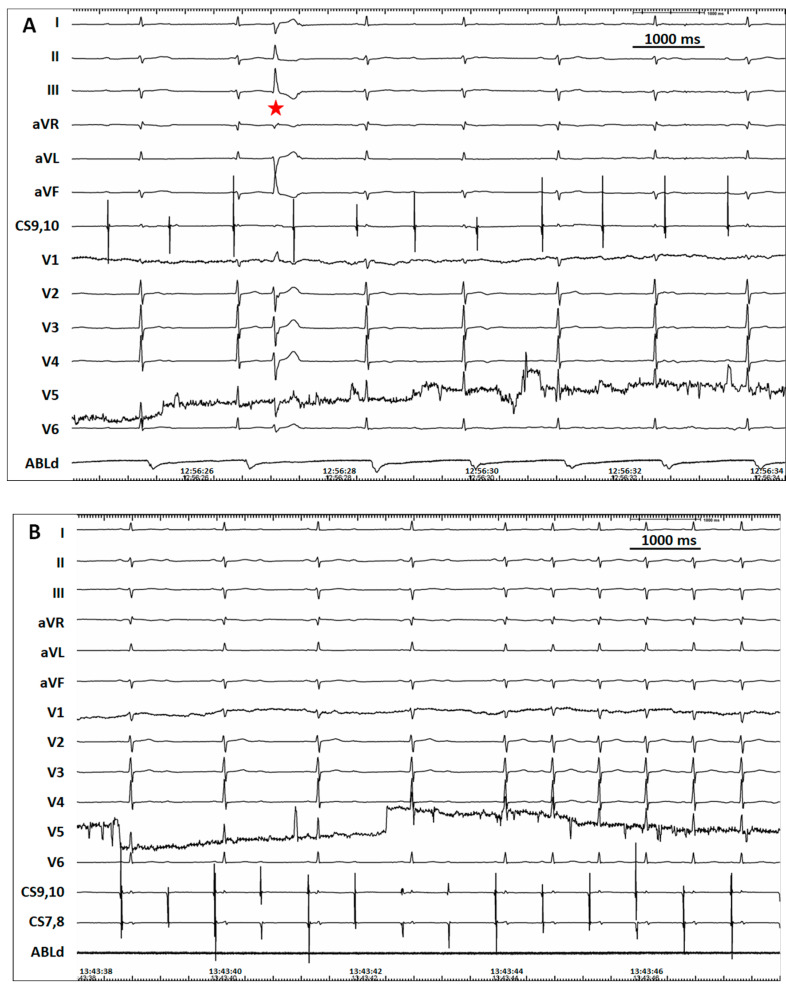
Atrioventricular (AV) block was encountered immediately when the ablation catheter was introduced into the left ventricle. (**A**). Infrequent premature ventricular complex (red star) remained while the AV conduction was completely blocked. (**B**). One-to-one AV conduction was gradually resumed after about 40 min of observation.

**Figure 3:**
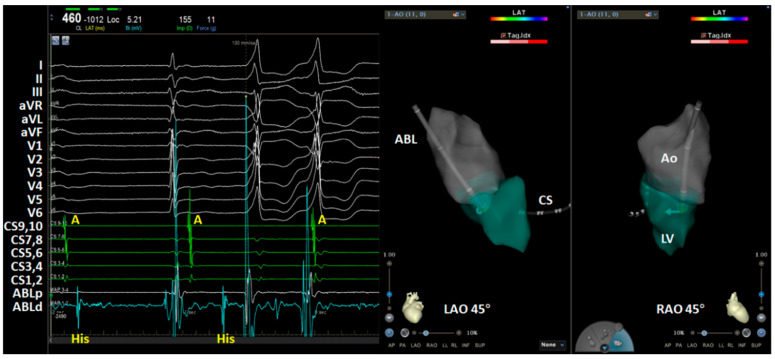
His potentials were recorded by the ablation catheter when the catheter was withdrawn from the left ventricle (LV). Note that the His potentials were dissociated with the atrial (A) and ventricular potentials. ABL, ablation catheter; Ao, aorta; CS, coronary sinus; LAO, left anterior oblique; RAO, right anterior oblique.

After written informed consent was obtained, an electrophysiology procedure was performed under the guidance of a three-dimensional electroanatomic mapping system (CARTO 3, Biosense Webster, Inc., Diamond Bar, CA, USA). A decapolar mapping catheter was placed within the coronary sinus via the right femoral vein. A 3.5 mm tip saline irrigating catheter (NaviStar ThermoCool SmartTouch, D curve, Biosense Webster) was used for mapping and ablation. After successful access to the right femoral artery, heparin was administrated. After the reconstruction of the ascending aorta, the activation mapping of the left ventricle was attempted by the retrograde transaortic approach. However, a complete AV block with junctional ectopy was encountered when the ablation catheter was introduced into the left ventricle with a “U” curve (“J” loop). She had no obvious symptom of bradycardia with the lowest heart rate of 38 bpm. Temporal ventricular pacing was not scheduled. The ablation catheter was withdrawn from the left ventricle. During the observation period, the PVC was also present but was infrequent (Figure 2A). About 30 min later, one-to-one conduction was resumed with the administration of dexamethasone, isoproterenol, and atropine. Another transaortic manipulation was attempted while the ablation catheter was directly introduced into the left ventricle in a very slight curve. However, a sustained complete AV block occurred again while the ablation catheter was crossing the aortic valve. Therefore, the ablation catheter was withdrawn under the circumstance of the AV block, and we found that the AV may be blocked at the AH and HV level (Figure 3). The AV conduction was recovered after a 40 min observation period (Figure 2B). After the resumption of AV conduction, no evidence of abnormality in the conduction system was revealed by an electrophysiological study (the AH interval was 103 ms, and the HV interval was 40 ms). A third retrograde transaortic approach was attempted; however, a complete AV block was encountered again. The procedure, which lasted for about 170 min, was abandoned considering the high risk of AV block. She did not encounter adverse sequalae during the overnight observation. A second procedure with the transseptal approach was proposed but refused by the patient and her relatives.

During a follow-up of 12 months, she remained with a normal AV conduction and frequent PVCs failed to be suppressed by antiarrhythmics. However, no severe dizziness or syncope was encountered by this patient, and no pacemaker implantation was needed.

## 3. Discussion

A retrograde transaortic catheter manipulation is a widely used and first-line access route for the mapping and ablation of ventricular arrhythmias arising from the left ventricular endocardium. Generally, the retrograde transaortic approach is safe. However, human anatomy studies have shown that the His-bundle penetrates the right fibrous trigone and emerges between the noncoronary cusp and right coronary cusp giving off the sheet of the left fascicles, which run down beneath the endocardium from the inferior ring of the membranous septum [5,6]. Therefore, a retrograde transaortic approach theoretically could lead to a mechanical AV block as a consequence of pressure exerted on the His–Purkinje system underneath the aorta. Usually, a mechanical AV block is transient and reversible as soon as the mechanical pressure is removed. However, the resumption of atrioventricular conduction after mechanical block may require a long time, as shown in this case. Therefore, a complete AV block is a rare but maybe life-threatening complication caused by the retrograde transaortic approach. 

A careful manipulation of the catheter to cross the aortic valve would reduce the incidence of a mechanical AV block caused by the retrograde transaortic approach. However, as presented in this case, a complete AV block was repeatedly encountered, even when manipulating the catheter with different curves or shapes to cross the aorta. Therefore, in some special patients, we should be cautious when a retrograde transaortic approach is attempted. It was reported that an extreme aortic root angulation with a horizontal aorta may present particular challenges for crossing the valve [3] and may cause a mechanical AV block in some patients. It would be more helpful to identify those patients with a high risk of mechanical AV block caused by the retrograde transaortic approach. However, currently, there are no sufficient clinical tools to identify patients at the highest risk for mechanical AV block caused by the retrograde transaortic approach. A transseptal approach would be the alternative access route for those patients encountering mechanical AV block caused by the retrograde transaortic approach. However, the transseptal approach needs a transseptal puncture, requires a long learning curve, and may increase the cost and procedure time. Moreover, available evidence indicates that the retrograde transaortic approach is superior for targeting basal inferior and lateral substrates, whereas the transseptal approach may be more effective for apical substrates [3]. Therefore, the retrograde transaortic approach is still the first-line access route for those arrhythmias arising from the left ventricular endocardium, especially for PVC originating from the left anterior fascicle [1,2,7,8]. 

In conclusion, a complete AV block due to a mechanical injury could occur during the introduction of a steerable ablation catheter to cross the aortic valve. We should be cautious when performing a retrograde transaortic catheter manipulation in some patients.

## Data Availability

The data presented in this study are available on request from the corresponding author.
